# Plötzliche bilaterale Visusminderung und Gesichtsfeldausfälle

**DOI:** 10.1007/s00347-021-01392-7

**Published:** 2021-04-29

**Authors:** Alexander C. Rokohl, Gerhard Welsandt, Ludwig M. Heindl, Friederike Schaub, Sigrid Roters

**Affiliations:** 1 Zentrum für Augenheilkunde, Medizinische Fakultät und Uniklinik Köln, Universität zu Köln, Kerpener Str. 62, 50937 Köln, Deutschland; 2 Medizentrum Porz, Hauptstr. 309, 51143 Köln, Deutschland

## Anamnese

Ein 14-jähriger Patient stellte sich notfallmäßig in der Universitäts-Augenklinik Köln vor und gab an, seit ungefähr 1 Tag beidseits einzelne Buchstaben beim Lesen nicht mehr richtig erkennen zu können. Er könne daher aktuell auch nicht mehr für seine Klassenarbeiten lernen. Die Augenanamnese war leer. Anamnestisch bestand nur ein leichtes allergisches Asthma ohne derzeitige Therapienotwendigkeit. Es bestanden keine körperlichen Beschwerden. Auch sonst waren keine weiteren Allgemeinerkrankungen, keine psychiatrischen Auffälligkeiten, keine B‑Symptomatik und auch kein Trauma bekannt. Der Patient nahm laut eigenen Angaben keinerlei Medikamente ein, und auch die Familienanamnese bezüglich ophthalmologischer Erkrankungen war leer.

Auf weitere Nachfrage gab der Patient an, einen Tag zuvor seine Katze mit dem Licht eines Laserpointers „gejagt“ zu haben. Teilweise hätte er auch den Laserstrahl an seinem Zimmerspiegel reflektieren lassen, um die Katze zu ärgern. Er hätte sich jedoch zu keinem Zeitpunkt direkt selbst in die Augen geleuchtet.

## Klinischer Befund

**Figure 1:**
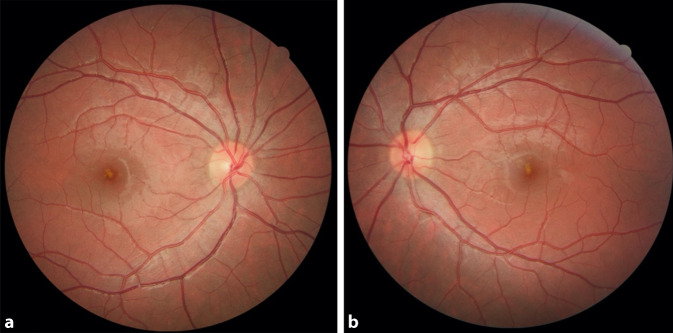

Der 14-jährige Patient befand sich in einem guten, altersüblichen Allgemein- und Ernährungszustand. Der Fernvisus lag initial mit bestmöglicher Refraktion (RA Sph −0,25 Cyl −0,25 A 6°; LA Sph −0,25 Cyl −0,25 A 41°) rechts bei 0,6 und links bei 0,5 dezimal. Der Augeninnendruck war beidseits mit 17 mm Hg normwertig. In der weiteren klinischen ophthalmologischen Untersuchung zeigte sich beidseits ein regelgerechter vorderer Augenabschnitt. Die Motilität beider Augen war frei, die Pupillen waren isokor, die direkte und indirekte Lichtreaktion seitengleich und prompt. Es lagen weder eine afferente Störung noch eine Rotentsättigung vor. Im Amsler-Gitter-Test zeigten sich beidseits parazentrale Skotome knapp unterhalb des Zentrums. Die Untersuchung der Netzhaut (Abb. 1a, b) zeigte bilateral einen vergrößerten fovealen Reflex und gelb-orange Läsionen im Bereich der Fovea.

## Weitere Diagnostik

**Figure 2:**
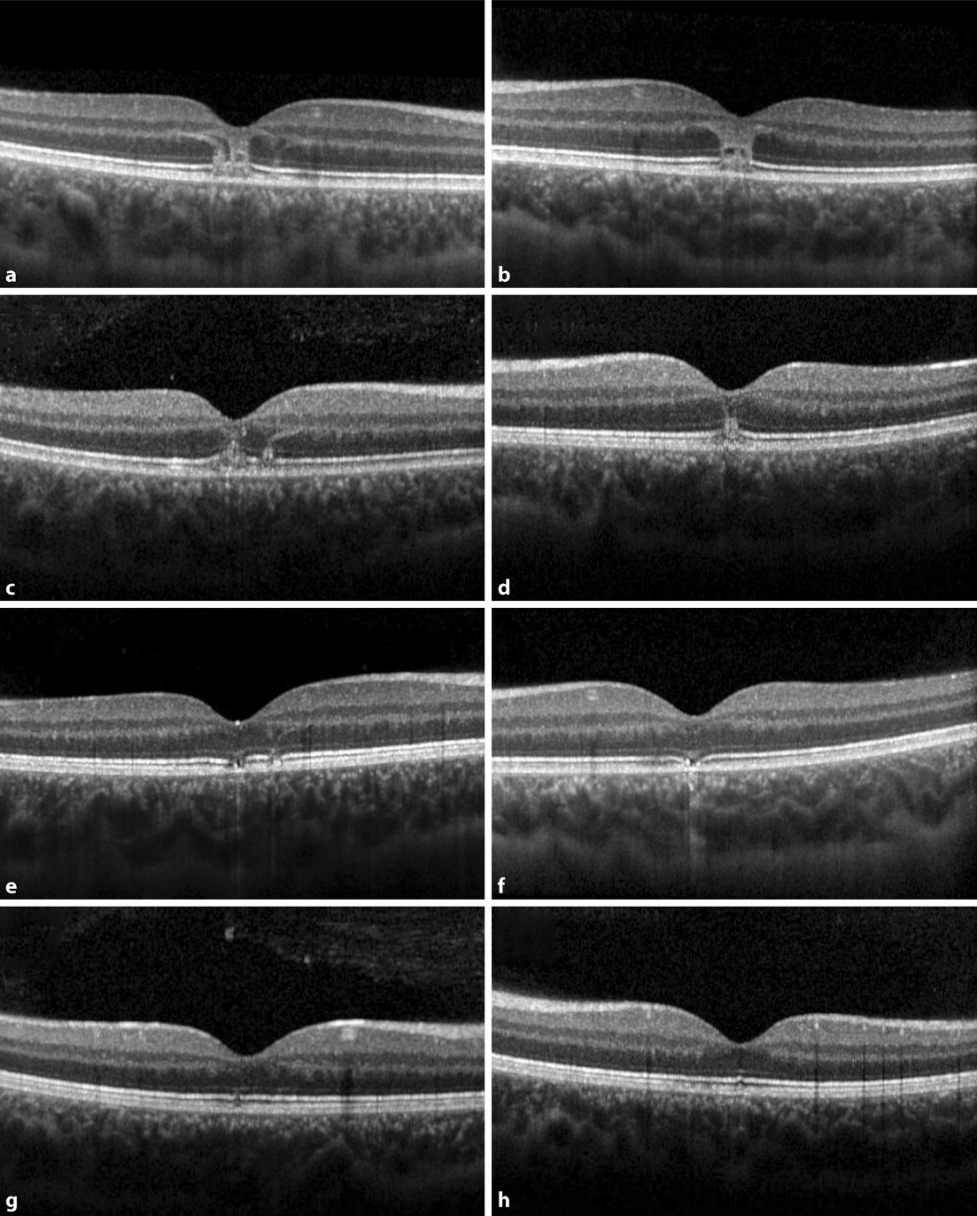

In der automatischen Computerperimetrie zeigten sich L > R multilokuläre, relative Skotome im 30°-Bereich, jedoch bei deutlich eingeschränkter Beurteilbarkeit, da dies die erste Gesichtsfelduntersuchung des jugendlichen Patienten überhaupt war. In der Spectral-Domain optischen Kohärenztomographie (SD-OCT) der Makula zeigten sich beidseits foveale hyperreflektive Läsionen der äußeren Netzhaut (von der äußeren plexiformen Schicht bis einschließlich der Fotorezeptoren) (Abb. 2a, b).

## Wie lautet Ihre Diagnose?

## Weitere Therapie und Verlauf

Im weiteren Verlauf und auf mehrfaches Nachfragen erzählte der Patient ehrlicherweise, dass er zwar nicht direkt in den Laserpointer geschaut hätte, aber er die Reflektion des Lasers in seinem Spiegel mehrmals deutlich wahrgenommen hätte.

Initial wurden eine systemische Hochdosissteroidtherapie mit Initialdosis Decortin H (1 mg/kgKG) sowie eine lokale Steroidtherapie (Inflanefran forte® [Allergan GmbH, Frankfurt am Main, Deutschland] Augentropfen 5‑mal täglich) beidseits ordiniert.

Drei Tage später war der bestkorrigierte Visus bereits rechts auf 0,63 und links auf 0,8 angestiegen bei bestem vorbekanntem Visus von beidseits 1,0. Zudem zeigte sich bereits eine Befundbesserung in der OCT (Abb. 2c, d), sodass die systemische Steroiddosis reduziert werden konnte.

**Figure 3:**
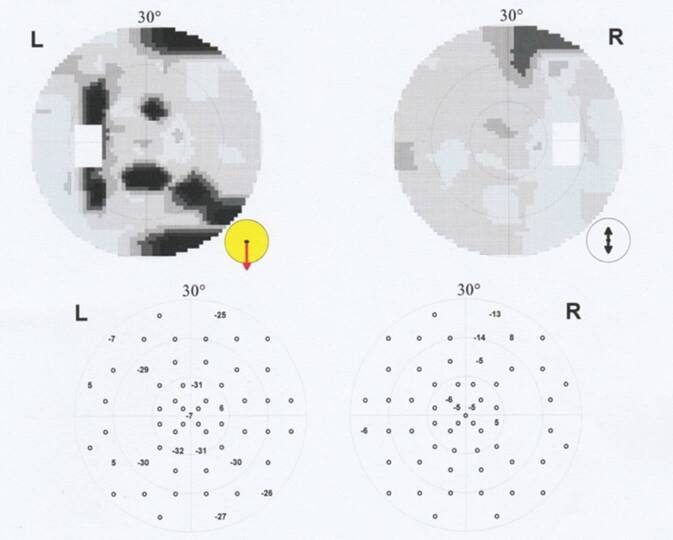

**Figure 4:**
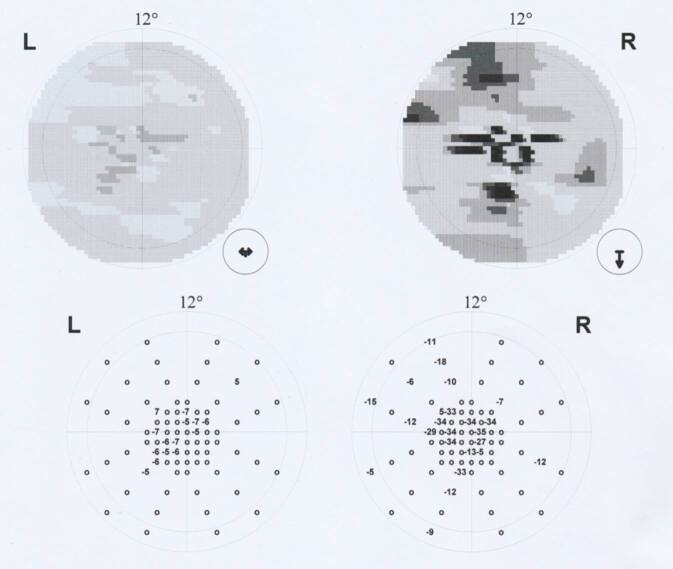

Einen Monat später zeigte sich unter ausschleichender Restdosis von 2,5 mg Decortin H systemisch und unter der Lokaltherapie mit Inflanefran forte® [Allergan GmbH, Frankfurt am Main, Deutschland] Augentropfen 5‑mal täglich ein weiterer Visusanstieg auf 1,0 beidseits bei persistierenden Skotomen im Amsler-Gitter-Test. Der Lesevisus betrug zu diesem Zeitpunkt nur 0,7 beidseits mit Birkhäuser-Tafeln bei einer deutlichen Verminderung der Lesegeschwindigkeit. Die morphologischen Defekte in der SD-OCT waren weiter rückläufig (Abb. 2e, f). Es zeigten sich lediglich noch Läsionen in der ellipsoiden Zone, den Fotorezeptoraußensegmenten und der interdigitalen Zone. In der automatischen Computerperimetrie bestanden zu dem Zeitpunkt links multilokuläre tiefe Skotome im 30°-Bereich, rechts dagegen multilokuläre relative Skotome (Abb. 3). Im 12°-Bereich waren jedoch auch rechts kleinere, multilokuläre tiefe Skotome nachweisbar (Abb. 4). Das Flicker-Gesichtsfeld wies bei jeder Untersuchung in der Zeit keinerlei Ausfälle auf.

Eine weitere Besserung bestand 2 Monate nach dem Ereignis mit stabilem vollem Fernvisus von 1,0 beidseits. Die auch subjektiv empfundenen „Löcher“ im Gesichtsfeld waren besser geworden; ebenso der OCT-Befund mit nur noch kleinen Unterbrechungen der ellipsoiden Zone und den Fotorezeptoraußensegmenten links > rechts.

Ein halbes Jahr nach der Exposition hatte der Patient keinerlei Probleme mehr bei stabilem Fernvisus 1,0. In der OCT zeigte sich nur noch eine kleine Unterbrechung der Fotorezeptoren zentral beidseits (Abb. 2g, h). Auch im 12°- und 30°-Gesichtsfeld waren nur noch schwache Relativskotome nachweisbar. Der Augendruck war zu jedem Untersuchungstermin normoton.

## Diskussion

Am häufigsten treten lichtbedingte Schädigungen der Netzhaut nach der Betrachtung einer Sonnenfinsternis auf [1]. Seltener können sie auch beim Sonnenbaden, beim Sonnenyoga oder bei religiösen Ritualen auftreten [1]. In den letzten Jahren scheint jedoch die Inzidenz der Laserpointer-assoziierten Retinopathien insbesondere bei jüngeren Patienten zu steigen [2–4]. Bei der Laserpointer-assoziierten Retinopathie kommt es zu einer extremen photothermischen Schädigung der Netzhaut, am ehesten vergleichbar mit einer Verbrennung, wobei insbesondere die Melanozyten des retinalen Pigmentepithels (RPE) und die benachbarten Photorezeptoren sehr vulnerable Zielstrukturen darstellen [2–6]. Die Strahlungsenergie wird v. a. im retinalen Pigmentepithel, das große Mengen von Melanin beinhaltet und das unter physiologischen Bedingungen als Lichtabsorber funktioniert, konzentriert [3, 5]. Das lokal begrenzte Aufheizen des Gewebes zieht dann eine Proteindenaturierung, einen Verlust der Zellintegrität und weitere inflammatorische Folgereaktionen nach sich [3]. Meist ist dabei die Fovea betroffen, da hier das Licht durch die Fixation fokussiert wird [3, 4].

Visuelle Symptome treten aufgrund der strukturellen Veränderungen durch die hochenergetische Laserstrahlung direkt nach der Exposition auf [1, 3, 4]. Typische Symptome beinhalten eine Visusminderung, Gesichtsfeldausfälle und verschwommenes Sehen [1, 3, 4]. Der Schweregrad der Visusbeeinträchtigung ist dabei sehr variabel und kann zwischen Fingerzählen und vollem Visus variieren [1, 3, 4].

Die Anamnese sollte bei Verdacht auf eine solare oder Laserpointer-induzierte Retinopathie bzw. Makulopathie immer eine ausführliche Evaluation der Begleitumstände beinhalten [2–4, 7]. Insbesondere sollte auch gefragt werden, ob der Laser ggf. an einem Spiegel oder anderen spiegelnden Flächen wie in unserem Fallbeispiel reflektiert worden sein könnte [7]. Da einige Studien zudem eine erhöhte Inzidenz selbstinduzierter Laserpointer-assoziierter Makulopathien bei psychisch auffälligen Kindern und Jugendlichen gezeigt haben, sollte auch dieser Umstand nicht außer Betracht gelassen werden [8, 9].

Die klinische Diagnostik sollte neben der Visusbestimmung für Ferne und Nähe auch eine Augeninnendruckmessung, einen Amsler-Gitter-Test, eine Testung der Lichtreaktion, eine Motilitätstestung sowie eine komplette spaltlampenmikroskopische Untersuchung inklusive einer Fundoskopie in Mydriasis beinhalten [2–4, 7–9]. Fundoskopisch präsentiert sich der Makuladefekt in der frühen Phase meist in Form eines gelblichen Fleckes umgeben von einem rötlichen Randsaum (Abb. 1; [1, 3]). Jedoch sind auch sehr variable Befunde wie Makulaforamina oder Netzhautblutungen möglich [3].

Größere Skotome können gut mit einer zentralen Standardperimetrie (z. B. 10-2 Gesichtsfeld) quantifiziert werden. Eine normale Standardperimetrie ist aufgrund der Ortsgenauigkeit von 1–2° jedoch nicht immer geeignet, um die Skotome bei der laserinduzierten Makulopathie nachzuweisen, da diese manchmal sehr klein sind (unter 1–2°) [1, 10]. Hier muss ggf. eine Mikroperimetrie hinzugezogen werden [1, 10].

Mittels multimodaler Bildgebung durch die OCT, die Fundusautofluoreszenz (FAF) sowie die Fluoreszeinangiographie (FAG) können die Läsionen detailliert detektiert und im Verlauf beurteilt werden [1, 3]. Oft zeigt sich in der OCT im Akutstadium neben einer Unruhe des retinalen Pigmentepithels – wie auch bei unserem Patienten – eine Hyperreflektivität der Netzhautschichten, ohne dass die Netzhautdicke beeinflusst ist [1, 3], jedoch einhergehend mit einer oftmals persistierenden Unterbrechung der äußeren Netzhautschichten [3].

Die Läsion stellt sich in der FAF meist hypofluoreszent dar und kann ggf. von einem hyperfluoreszenten Ring umgeben sein. In der Regel zeigt sich in der FAG eine Hyperfluoreszenz in der arteriovenösen Phase und in der Spätphase, ausgelöst durch einen Fensterdefekt.

Die therapeutischen Optionen von Laserpointer-assoziierten Makulopathien sind sehr limitiert [3]. Bislang fehlen systematische Therapiestudien, die eine funktionelle Verbesserung im Vergleich zum Spontanverlauf belegen. Meist zeigen Glukokortikoide jedoch eine positive Wirkung auf die sekundäre, lokale inflammatorische Komponente nach thermischer Schädigung der Netzhaut durch hochenergetische Laserstrahlung. Daher sollten Glukokortikoide, zumindest wenn kein anderer Grund dagegenspricht, möglichst sowohl topisch als systemisch gegeben werden, auch wenn das durch die thermische Einwirkung bereits in toto geschädigte Gewebe damit nicht erreicht wird.

Ein Makulaforamen verschließt sich zum Teil ohne Intervention; bei Persistenz kann evtl. chirurgisch ein Visusanstieg erreicht werden [3]. Intravitreale Injektionen von VEGF(„vascular endothelial growth factor“)-Inhibitoren können beim Auftreten einer sekundären CNV indiziert sein [3].

Bei unserem Patienten kam es im Verlauf aufgrund der eher leichten Makulopathie ohne signifikante Schädigung des retinalen Pigmentepithels zu einem Visusanstieg und zur Rückbildung der Skotome. Die genaue Prognose ist jedoch sehr individuell und abhängig vom Ausmaß und der Lokalisation der retinalen Schädigung sowie weiterer sekundärer Komplikationen [3]. Bei einem Teil der Patienten zeigen sich persistierende Visusminderungen und Skotome, die zu dauerhaften Beeinträchtigungen im Alltag bis hin zur Berufsunfähigkeit oder Erblindung führen können [3, 10].

**Diagnose:** Bilaterale, Laserpointer-induzierte Makulopathie durch Reflexion an einem Spiegel

Mögliche Differenzialdiagnosen umfassen v. a. Makuladystrophien sowie entzündliche und ischämische Retinopathien, die Poppers-Makulopathie, die luesassoziierte Makulopathie, eine Tamoxifen-Makulopathie oder einen Morbus Stargardt [3] und sollten anamnestisch oder differenzialdiagnostisch bei unklarer Ursache ausgeschlossen werden.

Die zunehmende Inzidenz der laserinduzierten Makulopathie beruht zum Teil auf einem relativ einfachen Zugang über den Onlinehandel zu nicht zertifizierten Laserpointern, die gerne als Spielzeug deklariert werden [3]. Zudem haben diese Geräte meist auch eine deutlich höhere Leistung als angegeben [3, 9]. Zu guter Letzt kann auch ein fehlendes Gefahrenbewusstsein dazu beitragen – insbesondere bei Jugendlichen [3]. Eine gute Aufklärungs- und Präventionsarbeit ist daher unerlässlich.

## Fazit für die Praxis

Zusammenfassend können Laserpointer bei nicht sachgemäßer Anwendung und auch durch eine Reflexion an einer spiegelnden Oberfläche für das Auge eine ausgeprägte Gefahr darstellen. Die Inzidenz der Laserpointer-induzierten Makulopathie scheint in den letzten Jahren zu steigen. Typischerweise klagen die Patienten über eine plötzliche Sehverschlechterung und über zentrale Skotome. In der Diagnostik und in der Verlaufsbeurteilung spielt die multimodale Bildgebung eine entscheidende Rolle. Teilweise bildet sich die Symptomatik bei milden Affektionen innerhalb von einigen Wochen zurück, jedoch bleiben oft irreversible Visusminderungen und Skotome bestehen, die dauerhafte Beeinträchtigungen im Alltag bis hin zur Berufsunfähigkeit oder Erblindung nach sich ziehen können. Da eine evidenzbasierte Therapie bisher fehlt und die Folgeschäden gravierend sein können, stehen die Prävention und die Aufklärung mit einer Schärfung des gesellschaftlichen Bewusstseins im Vordergrund.
