# Erratum zu: *Plötzliche bilaterale Visusminderung und Gesichtsfeldausfälle*

**DOI:** 10.1007/s00347-021-01424-2

**Published:** 2021-05-20

**Authors:** Alexander C. Rokohl, Gerhard Welsandt, Ludwig M. Heindl, Friederike Schaub, Sigrid Roters

**Affiliations:** 1 Zentrum für Augenheilkunde, Medizinische Fakultät und Uniklinik Köln, Universität zu Köln, Kerpener Str. 62, 50924 Köln, Deutschland; 2 Medizentrum Porz, Köln, Deutschland

**Erratum zu:**

**Ophthalmologe 2021**

10.1007/s00347-021-01392-7

In dem ursprünglichen Artikel wurden die beiden Teilabbildungen 1 a und b vertauscht. Die Online-Version sowie das PDF des Beitrags wurden nachträglich korrigiert. Bitte beachten Sie die korrigierte Abbildung (Abb. 1).

**Figure 1:**
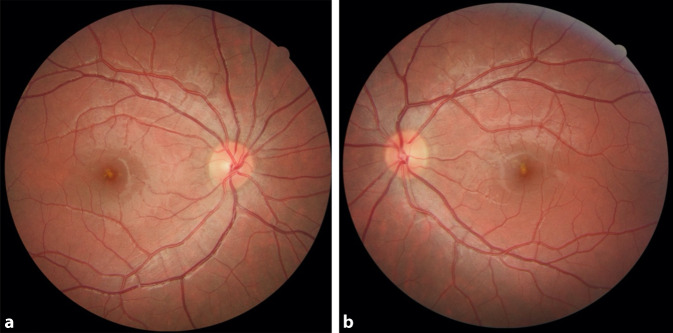

Der vollständige und korrigierte Artikel steht Ihnen auf www.springermedizin.de zur Verfügung. Bitte geben Sie dort den Beitragstitel in die Suche ein.

Die Redaktion

